# Factors affecting seeking psychological and psychiatric support for Turkish society

**DOI:** 10.1371/journal.pone.0310982

**Published:** 2024-09-25

**Authors:** Kübranur Çebi Karaaslan, Abdulkerim Karaaslan, Hüriye Subaşı

**Affiliations:** 1 Faculty of Economics and Administrative Sciences/Economic and Social Research Application and Research Center, Department of Econometrics, Erzurum Technical University, Yakutiye/Erzurum, Türkiye; 2 Faculty of Economics and Administrative Sciences, Department of Econometrics, Atatürk University, Türkiye, Master Research Training and Consulting Services Ltd. Şti., Ata Teknokent, Erzurum, Türkiye; 3 Department of Office Services and Secretary Vocational School of Social Sciences Ataturk University, Erzurum, Türkiye; Universitas 17 Agustus 1945 - Jakarta, INDONESIA

## Abstract

The increase in mental health issues and awareness among individuals, compared to previous times, has contributed to recognizing this as a significant public health issue. The necessity to explore potential factors behind mental health problems has become paramount and served as the primary impetus for the current research endeavor. This study aims to explore if there is a relationship between attitudes toward seeking psychological and psychiatric support. a significant relationship between sociodemographic factors and individuals’ tendency to seek psychological and psychiatric help, and if so, in what direction are these relationships? and a significant relationship between individual and social factors and individuals’ tendency to seek psychological and psychiatric help, and if so, in what direction are these relationships? Microdata from the Turkey Health Survey, conducted by the Turkish Statistical Institute and representative of Turkey, was used as the dataset. Increased the representative power of the data set over the universe by weighting the data set, and the Bivariate Probit Model was used to address the research questions. As a result of the study, various sociodemographic (gender, age, employment status, et al.), individual and social factors (general health status, participation in social activities, alcohol use et al.) may influence individuals’ inclinations toward seeking psychological and psychiatric assistance. The research offers valuable insights for social policymakers and researchers into the behavioral tendencies of individuals seeking professional psychiatric help.

## Introduction

The mental well-being of individuals plays a pivotal role in determining their level of life satisfaction, measured by the overall evaluation of one’s quality of life and the extent of positivity. In essence, a favorable psychological state contributes to individuals’ ability to experience joy in their lives. Consequently, they are likely to experience happiness, contentment, and positive life outcomes [1–3]. Due to the heightened expectations, discontent, and perpetual rush prevalent in today’s world, individuals are grappling with psychological distress, necessitating interventions to enhance and support their mental well-being [4]. Paradoxically, a significant number of individuals resist seeking psychological support despite these challenges [5]. Notably, those with elevated levels of depression exhibit a notably low inclination to seek such assistance [6–8]. The willingness to seek psychological support appears to be associated with personality-related emotional issues, as opposed to economic problems [9,10]. The initiation of the process of seeking psychological support hinges on the recognition of a problem [11]. However, disbelief in the existence of an issue or an oversight in acknowledging its intertwining with personal challenges becomes a hindrance, deterring individuals from seeking the necessary support. This discrepancy highlights that the perceived need for psychological support and the actual receipt of support may not align in actuality. The extensive literature that exists in this field indicates that various factors, including demographic, individual, and environmental elements, exert influence on the inclination to seek psychological support [11–19].

Chen et al. (2020) investigated the attitudes and factors influencing the pursuit of professional psychological assistance. Multiple linear regression analysis showed that gender, age, employment status, depression literacy, stigma, and help-seeking intention are significantly associated with attitudes toward seeking professional psychological help [20]. Husain (2020) aimed to explore the barriers that prevent from seeking psychological help. Data was collected through interviews and a self-report questionnaire. Barriers to seeking psychological help included lack of faith in psychological treatment, prior personal experience, religious fatalism, carelessness towards mental disorders, fear of social stigma, personal shame, negative perceptions of mental health practitioners, opposition from family, and fear of treatment [21]. Molla (2021) studied students’ academic self-concept, their behaviors in seeking academic help, and their beliefs regarding the effectiveness of counseling services. An independent-sample t-test revealed that male students had significantly higher average scores than female students in academic self-concept, help-seeking behavior, and belief in counseling effectiveness [22]. Ülken and Odacı’s study (2021) involving 786 university students identified significant differences in psychological support-seeking attitudes based on gender, faculty, and place of residence, while no significant differences were found based on income level [23]. Dalum et al. (2022) investigated the prevalence of mental health problems and professional help-seeking behavior for such problems, as well as the independent factors associated with help-seeking behavior among veterinarians. Logistic regression was used to analyze professional help-seeking for mental health problems. Analyses were controlled for socio-demographic, individual, and work-related factors. Thirty percent of veterinarians in Norway reported experiencing mental health issues requiring treatment, but only half of them had sought professional help. A low level of help-seeking behavior was also observed among those who had serious suicidal thoughts. Factors such as being female, having positive attitudes toward mental health treatment, and working in public administration, academia/research, or other fields were associated with higher rates of seeking help. Conversely, working in production animal practice was associated with lower rates of seeking help [24].

Gearing et al. (2022) of research show that people’s attitudes toward seeking professional help for mental health are influenced by knowing someone with a mental health issue. Additionally, older men who were married and had children were more open to seeking help for mental health, highlighting the significance of life experiences in shaping attitudes toward seeking help [25]. Nagai (2022) explored the connections between three help-seeking indices—help-seeking intentions, help-seeking attitudes, and help-seeking styles—and two aspects of gender role conflict: restrictive emotionality and success/power/competition. The study indicates that although gender role conflict is correlated with attitudes toward help-seeking, its impact on help-seeking behavior is minimal [26]. A Structural Equation Modelling approach was used to study the effects of distress, stigma, and coping strategies on professional help-seeking intentions. Dagani et al. (2023) study suggests that students with significant psychological distress use coping strategies to face the stigma of seeking help and the lower the stigma of seeking help, the higher the chance of developing intentions to seek professional help [27]. Novak et al. (2023) examined help-seeking among individuals admitted for psychiatric inpatient care. Hierarchical binary logistic regressions were used to examine associations among types of helping resources, mental health treatment stigma, and perceived social support. About 90% of the participants sought help before being hospitalized, with behavioral health providers and friends being the most frequently accessed resources. The resources that were accessed were generally considered helpful. When adjusting for other factors, it was found that stigma related to mental health treatment was not linked to seeking help from any specific type of resource. However, higher perceived social support was associated with a greater likelihood of seeking help from a friend. It was also found that marital status and education level were linked to not seeking help [28]. Baklola et al. (2024) assessed the levels of Mental health literacy and help-seeking behavior of individuals. The findings advocate for comprehensive mental health education, particularly in rural areas, and emphasize the role of personal relationships in mental well-being [29].

Mental well-being, a crucial part of overall well-being, involves an individual’s ability to cope with daily life stresses, contribute effectively to work-related tasks, and actively engage in society [29]. The fact that mental well-being is a significant component of overall health, which has no alternative and is the most important necessity in life, constitutes the motivation for the present study. Thus, the present study aims to determine the factors affecting individuals’ attitudes toward seeking psychological support and the aspects and dimensions of the impact of these factors. The study is of importance in terms of individuals who require psychological support and those who do not seek support even though they need it, as well as raising awareness for those who are not particularly willing to receive support due to specific issues. In this context, the original aspects of the study could be as the examination of the attitudes towards both seeking psychological and psychiatric support in conjunction with each other through a data set representative of Türkiye in a manner that has been not in the literature up to date, the use of a very large sample by weighting the data set, and the utilization of the Bivariate Probit Model, an advanced statistical method. It is anticipated that the study will make substantial contributions to the literature.

The research questions this study focuses on are as follows:

Research Question 1: Is there a relationship between attitudes towards seeking psychological and psychiatric support?

Research Question 2: Is there a significant relationship between sociodemographic factors and individuals’ tendency to seek psychological and psychiatric help, and if so, in what direction are these relationships?

Research Question 3: Is there a significant relationship between individual and social factors and individuals’ tendency to seek psychological and psychiatric help, and if so, in what direction are these relationships?

## Methods

### Data source

The present study utilizes the dataset of the 2022 Türkiye Health Survey conducted by the Turkish Statistical Institute. All settlements within the borders of the Republic of Türkiye constitute the geographical boundaries of the survey and the study was designed to yield an overall estimate for Türkiye. Stratified Two-Stage Cluster Sampling was employed as the sampling method. The framework used in the sample study of the research is the "National Address Database", which was updated in August 2022. Weighting coefficients calculated through the current population projections according to the Address-Based Population Registration System were used, thus increasing the representative power of the data set over the universe by weighting the data set. In the survey conducted by the official institution, the data set of individuals over the age of 15 consists of 22742 observations. The entire data set was used in the analyses, without any missing observations [30]. The data underlying this study is subject to third-party restrictions by the Türkiye Statistical Institute. Data are available from the Turkish Statistical Institute (bilgi@tuik.gov.tr) for researchers who meet the criteria for access to confidential data.

### Outcome variables

The dependent variables used in the present study are the individuals’ visits to psychologists and psychiatrists. These variables were measured with the questions "Have you visited a psychologist for yourself in the last 12 months?" and "Have you visited a psychiatrist for yourself in the last 12 months?".

### Explanatory variables

The socio-demographic, individual, and social factors that could potentially affect individuals’ attitudes toward seeking psychological support were included in the model as explanatory variables. The variables of age, gender (male, female), educational status (not graduated, primary school, secondary school, high school, university), marital status (never married, married, divorced-widowed), and employment status (employed, unemployed) are the sociodemographic factors.

**Fig 1:**
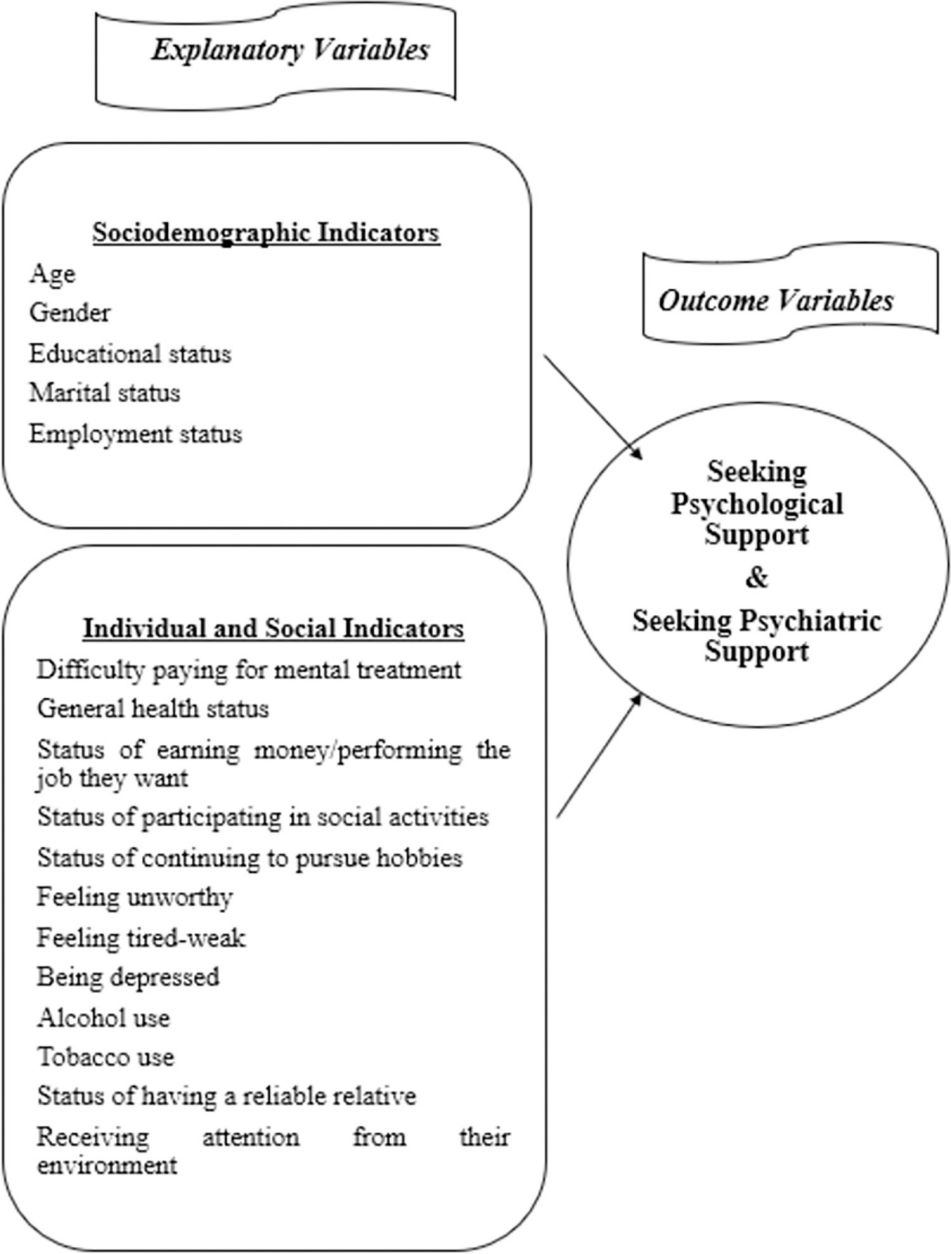
Research model.

The variables of difficulty paying for mental treatment (yes, no, did not need it), general health status (very good-good, moderate, bad-very bad), status of earning money/performing the job they want (no difficulty, some difficulty-great difficulty, cannot perform at all, not interested-does not want to perform it), status of participating in social activities (no difficulty, some difficulty-great difficulty, cannot participate at all, not interested-does not want to participate), status of continuing to pursue hobbies (no difficulty, some difficulty-great difficulty, cannot pursue at all, not interested-does not want to pursue), feeling unworthy (never, in certain days-more than a week-almost every day), feeling restless (never, in certain days-more than a week-almost every day), feeling tired-weak (never, in certain days-more than a week-almost every day), being depressed (yes, no), alcohol use (yes, no), tobacco use (yes, every day-yes, occasionally, quit, never used), status of having a reliable relative (none, 1–2 people-3-5 people-6 or more people) and receiving attention from their environment (a lot-high-moderate-little, none) are the individual and social factors. Due to the presence of low-frequency observations in certain categories of the explanatory variables, category combinations were made. The independent and dependent variables of the study are associated according to the research model shown in Fig 1.

### Statistical analysis

In this study, which is binary. For this reason, the Bivariate Probit Model was used in the analysis of the data set.

The Bivariate Probit model extends the capabilities of the logistic regression model, offering a more comprehensive approach to modeling relationships. The Bivariate Probit Model is a joint model used for two bivariate dependent variables whose distributions are assumed to be correlated. To use this model, dependent variables must be related. The Bivariate Probit Model is a joint model used for two bivariate dependent variables whose distributions are assumed to be correlated and it is assumed that error terms are drawn from a standard bivariate normal distribution with zero means, unit variances, and correlation coefficient ρ. The coefficients can be estimated using the maximum likelihood estimation. After estimating the parameters, it has to consider the marginal effects of the covariates in the conditional distribution for the interpretation of the coefficient. Marginal effects determine the magnitude of change of the conditional probability of the outcome variable when you change the value of a regressor, holding all the regressors constant at some value The marginal effects for categorical variables demonstrate how the conditional probability changes as the categorical variable changes from 0 to 1, after controlling for the other variables in the model [31].

In this study, Microsoft Excel was used to arrange the data set while Stata 16 was used for analysis. In the Bivariate Probit Model constructed within the study, the dependent variable categories take the value of 1 if the individual seeks psychological support, 0 if the individual does not seek psychological support, 1 if the individual seeks psychiatric support, and 0 if the individual does not.

## Results

### Characteristics of study participants

Table 1 shows the descriptive statistics regarding the factors that could influence individuals’ tendency to seek professional psychological support.

**Table 1:** Descriptive statistics of study participants.

Variables		N	% or μ (sd)
**Sociodemographic Indicators**			
Age		22742	43.37 (17.52)
Gender			
	Male	10971	48.24
	Female	11771	51.76
*Educational Status*			
	Not Graduated	2369	10.42
	Primary School	6848	30.11
	Secondary School	3853	16.94
	High School	5063	22.26
	University	4609	20.27
*Marital Status*			
	Never Married	5467	24.04
	Married	15049	66.17
	Divorced-Widowed	2226	9.79
*Employment Status*			
	Employed	9069	39.88
	Unemployed	13673	60.12
**Individual and Social Indicators**			
*Difficulty Paying*			
	Yes	1842	8.10
	No	19026	83.66
	Did Not Need It	1874	8.24
*General Health Status*			
	Good	14204	62.46
	Moderate	6680	29.37
	Bad	1858	8.17
*Earning Money/Performing the Job Desired*			
	No Difficulty	15725	69.15
	Some Difficulty	1016	4.47
	Cannot Perform	526	2.31
	Not Interested-Does Not Want to Perform	5475	24.07
*Participation in Social Activities*			
	No Difficulty	20124	88.49
	Some Difficulty	1110	4.88
	Cannot Participate	370	1.63
	Not Interested-Does Not Want to Participate	1138	5.00
*Continuing to Pursue Hobbies*			
	No Difficulty	19445	85.50
	Some Difficulty	1021	4.49
	Cannot Pursue	446	1.96
	Not Interested-Does Not Want to Pursue	1830	8.05
*Feeling Unworthy*			
	Not at All	18919	83.19
	Yes	3823	16.81
*Feeling Restless*			
	Not at All	20472	90.02
	Yes	2270	9.98
*Feeling Tired-Weak*			
	Not at All	12.716	55.91
	Yes	10.026	44.09
*Being Depressed*			
	Yes	1637	7.20
	No	21105	92.80
*Alcohol Use*			
	No	18841	82.85
	Yes	3901	17.15
*Tobacco Use*			
	Yes	7280	32.01
	Quit	2129	9.36
	Never Used	13333	58.63
*Having a Reliable Relative*			
	No	923	4.06
	Yes	21819	95.94
*Attention from the Environment*			
	Yes	21605	95.00
	No	1137	5.00

In terms of the sociodemographic variables, the mean age of the individuals was 43 years, 48.24% were male, 20.27% were university graduates, 66.17% were married, and 39.88% were employed. In terms of the individual and social indicators, 62.46% of the individuals had good general health, 69.15% had no difficulty in earning money, 88.49% had no difficulty in participating in social activities, 85.50% had no difficulty in pursuing their hobbies, 16.81% feel unworthy, 9.98% feel restless, 44.09% feel weak-tired, 7.20% are depressed, 17.15% use alcohol, 32.01% use tobacco, 4.06% have no reliable relatives and 5% do not receive attention from their environment.

### Findings on model estimations

Due to the categorical nature of the dependent variables, a Bivariate Probit Model was used in the present study and the variance inflation factors were examined for the analysis of multicollinearity among the explanatory variables included in the model. Variance inflation factors being below 5 indicates that there is no multicollinearity. Table 2 shows the model estimation results regarding the factors that could influence the individuals’ attitudes toward seeking psychological and psychiatric support through weighted data.

**Table 2:** Bivariate Probit Model estimation results regarding seeking psychological support and seeking psychiatric support.

Variables		Seeking Psychological Support			Seeking Psychiatric Support		
		β	Std. Error	*p*	β	Std. Error	*p*
**Sociodemographic Indicators**							
*Age*		-0.007	0.002	0.001	-0.006	0.002	0.001
*Gender (reference*: *male)*							
	Female	0.192	0.050	0.000	0.196	0.049	0.000
*Educational Status (reference*: *not graduated)*							
	Primary School	0.252	0.080	0.002	0.206	0.074	0.005
	Secondary School	0.346	0.095	0.000	0.225	0.086	0.009
	High School	0.342	0.093	0.000	0.254	0.082	0.002
	University	0.409	0.094	0.000	0.322	0.086	0.000
*Marital Status (reference*: *married)*							
	Never Married	-0.052	0.061	0.392	-0.087	0.060	0.149
	Divorced-Widowed	0.212	0.065	0.001	0.126	0.062	0.041
*Employment Status (reference*: *unemployed)*							
	Employed	-0.056	0.051	0.268	-0.077	0.051	0.127
**Individual and Social Indicators**							
*Difficulty Paying (reference*: *no)*							
	Yes	0.017	0.067	0.805	0.038	0.063	0.543
	Did Not Need It	-0.158	0.106	0.136	-0.293	0.105	0.005
*General Health Status (reference*: *bad)*							
	Good	-0.287	0.081	0.000	-0.236	0.078	0.002
	Moderate	-0.157	0.071	0.027	-0.085	0.069	0.219
*Earning Money/Performing the Job Desired (reference*: *no difficulty)*							
	Some Difficulty	0.018	0.094	0.844	0.013	0.087	0.879
	Cannot Perform	0.051	0.134	0.706	0.254	0.113	0.025
	Not Interested-Does Not Want to Perform	-0.045	0.057	0.428	0.032	0.052	0.545
*Participation in Social Activities (reference*: *no difficulty)*							
	Some Difficulty	0.197	0.112	0.078	0.044	0.091	0.630
	Cannot Participate	0.482	0.157	0.002	0.262	0.147	0.074
	Not Interested-Does Not Want to Participate	0.103	0.111	0.352	-0.088	0.108	0.414
*Continuing to Pursue Hobbies (reference*: *no difficulty)*							
	Some Difficulty	-0.076	0.116	0.511	0.004	0.095	0.967
	Cannot Pursue	-0.183	0.172	0.286	-0.273	0.174	0.116
	Not Interested-Does Not Want to Pursue	0.097	0.090	0.280	0.072	0.084	0.394
*Feeling Unworthy (reference*: *not at all)*							
	Yes	0.070	0.070	0.318	0.171^a^	0.063	0.007
*Feeling Restless (reference*: *not at all)*							
	Yes	-0.037	0.067	0.581	0.080	0.065	0.217
*Feeling Tired-Weak (reference*: *not at all)*							
	Yes	0.061	0.050	0.219	0.027	0.046	0.568
*Being Depressed (reference*: *no)*							
	Yes	0.979	0.053	0.000	1.336	0.050	0.000
*Alcohol Use (reference*: *yes)*							
	No	-0.155	0.056	0.006	-0.135	0.053	0.011
*Tobacco Use (reference*: *never used)*							
	Yes	0.022	0.075	0.764	0.117	0.077	0.129
	Quit	-0.032	0.075	0.672	-0.048	0.077	0.534
*Having a Reliable Relative (reference*: *yes)*							
	No	-0.065	0.091	0.476	-0.068	0.089	0.445
*Attention from the Environment (reference*: *no)*							
	Yes	-0.038	0.085	0.658	-0.193	0.078	0.013
*Constant Term*		-1.943	0.213	0.000	-1.792	0.208	0.000
*RHO*		0.681	0.038	0.000			

Likelihood-ratio test RHO = 0: χ2 (1) = 373.116 p> χ2 = 0.00001.

According to the results of the likelihood-ratio test performed to determine the significance of the RHO coefficient measuring the correlation between the errors as shown in Table 2, it was determined that the models constructed for the attitudes towards seeking psychological and psychiatric support are positively correlated with each other and that it is more suitable to use a Bivariate Probit Model simultaneously instead of two separate univariate probit models (RHO = 0.68; *p*<0.0001) [32,33].

The model estimate was found to be statistically significant. Following the model estimation, coefficient interpretations will be performed through marginal effects.

According to the marginal effects of the covariates, the likelihood of both receiving psychological support and psychiatric support decreased as the age of the individual increased. The women were found to be 0.40% more likely to receive both psychological support and psychiatric support compared to the men. Being a primary school, secondary school, high school, and university graduate increased the likelihood of receiving both psychological support and psychiatric support by 0.37%, 0.51%, 0.53%, and 0.70% compared to the reference group. Divorced-widowed individuals are 0.43% more likely to receive both psychological and psychological support compared to married individuals. The individuals who are employed were found to be 0.13% less likely to receive both psychological and psychiatric support compared to the unemployed individuals.

Individuals who have not needed medical care in the last 12 months are 0.36% less likely than those who need medical care but have difficulty paying to seek both psychological and psychiatric support. Those with good and moderate general health are 0.62% and 0,33% less likely to seek both psychological and psychiatric support compared to the reference group, respectively. An individual who does not engage in social activities is 1.14% more likely to seek both psychological and psychiatric support compared to those who do not face difficulties in participating in these activities.

Individuals who feel unworthy were found to be 0.37% more likely to seek both psychological and psychiatric support compared to those who do not feel unworthy. Individuals who are depressed are 5.98% more likely to seek both psychological and psychiatric support in comparison with those who are not depressed. Individuals who do not use alcohol are 0.34% less likely to seek both psychological and psychiatric support compared to those who use alcohol.

## Discussion

The increase in mental health problems and the growing awareness among individuals in recent times have led to the recognition of this issue as a major public health concern. It is now imperative to investigate the possible factors contributing to this rise in mental health issues. This concern has been the main driving force behind the ongoing research efforts. Within the scope of the present study, the factors affecting individuals’ attitudes toward seeking both psychological and psychiatric support were examined through a Bivariate Probit Model. The anticipated outcomes of this study hold the potential to provide valuable insights for researchers engaged in psychological research and inform social policymakers in their decision-making processes.

It was found that individuals’ likelihood of seeking both psychological and psychiatric support decreases as they age. Activities aimed at improving mental health, particularly for adolescents who will shape the future, have the potential to yield positive outcomes for both these individuals and the surrounding society as a whole throughout their lives. In the literature, studies are reporting that older individuals have a more positive approach towards seeking psychological support [5,34–37], a study that found no correlation between age and seeking psychological support [38], as well as studies that identified a negative correlation between age and the attitude towards seeking psychological support [39].

Women have a higher probability of seeking both psychological and psychiatric support compared to men. Studies are reporting similar findings in the literature [5,7,24,40–43]. This situation may be attributed to the probability that women are more open to identifying and solving their psychological problems and receiving help for these problems in comparison to men, whereas men may perceive this as a weakness [41]. In the study conducted by Boyd et al. [2011] on adolescents, it was found that males residing in rural areas tended to seek more psychological support compared to their female counterparts [44].

It has been that individuals’ likelihood of seeking both psychological and psychiatric support increased in parallel with their education level. Studies are reporting similar findings in the literature [21,28,39]. This situation may be associated with the idea that educated individuals may have a higher level of awareness with the view that education is a journey for self-development, and as a result, they may better assess their situation and not hesitate to seek psychological support.

Working individuals are less likely to seek both psychological and psychiatric support compared to unemployed individuals. Supporting this finding, a study has been conducted [21]. Within the framework that this is an economic indicator, there are previous studies in the literature reporting that the tendency to seek psychological support decreases as income level increases [5,39,45]. This is an anticipated conclusion. Furthermore, within the framework of the idea that happy individuals require less psychological support compared to unhappy individuals, numerous studies in the literature determined that there is a positive correlation between income level and happiness [46–59] and that working individuals are happier compared to unemployed individuals [57,60–62].

Individuals who have not needed medical care in the last 12 months are less likely than those who need medical care but have difficulty paying to seek both psychological and psychiatric support. Similarly, individuals with good general health are less likely to seek both psychological and psychiatric support compared to those with poor health. Psychological well-being may be a component of being healthy, and likewise, being healthy may have a positive effect on the psychology of individuals and lead to psychological well-being. In support of this argument, a considerable number of studies report that healthy individuals experience more happiness in life [53,54,62–65]. Additionally, in a previous study investigating the sources of happiness, which can be defined as a state of psychological well-being, it was determined that the most significant factor for happiness is physical health [66].

Individuals who do not have any income are more likely to seek both psychological and psychiatric support compared to those who do not experience financial difficulties. This may be related to employment, within the framework that individuals who do not experience financial difficulties are those who are already employed while individuals who do not have an income are unemployed. Thus, working individuals may be happier than those who are unemployed. In fact, studies have reported that working individuals are happier than unemployed individuals [37,60–62].

Individuals who use alcohol are more likely to seek both psychological and psychiatric support compared to those who do not use alcohol. Divorced-widowed individuals are more likely to receive both psychological and psychological support compared to married individuals. Some studies in the literature support these results [67,68].

An individual who does not engage in social activities such as meeting with family or friends, dining out, or participating in social events [such as going to the movies or watching a match, etc.] is more likely to seek both psychological and psychiatric support compared to those who do not face difficulties in participating in these activities. There is a significant role of personal relationships in mental well-being [29]. There is a study in the literature reporting that support from family and social environments positively affects the likelihood of receiving psychological support [37].

Individuals experiencing feelings of unworthiness are more likely to seek both psychological and psychiatric support compared to those who do not feel unworthy. Individuals who are depressed are also more likely to seek both psychological and psychiatric support in comparison with those who are not. This also applies to those who tend to seek professional support [29]. The specific psychological problems experienced by individuals are an important factor in the process of deciding to seek psychological support. Moreover, there are previous studies in the literature that have identified significant relationships between psychological distress and seeking psychological support [8,41,69–71]. Additionally, in parallel with these results, the area exhibiting the highest level of inclination to seek psychological support is personality-related problems [9].

It was found that individuals’ age, gender, education level, marital status, employment status, satisfaction with their health, financial difficulties, participation in social activities, feelings of unworthiness, depression, and alcohol use had an impact on their attitudes toward seeking psychological and psychiatric support. Mental health is an essential component of overall health. Therefore, the findings of the present study are of significance.

A nationally representative sample of the country was used in this study, which examined the factors affecting individuals’ attitudes toward seeking both psychological and psychiatric support. Increased the representative power of the data set over the universe by weighting the data set, and the Bivariate Probit Model was used to address the research questions. This study has several limitations. The data of the study are secondary data. Since the data is cross-sectional, a definitive causal relationship cannot be established. The variables required for statistical analysis are variables that are in the data set and can be created by researchers through the data set.

## Conclusion

Despite cultural differences, mental health services are underutilized worldwide due to various psychosocial barriers. The primary obstacle is the fear of being stigmatized, which prevents people from seeking help. Help-seeking behavior is complex and influenced by many factors. Encouraging help-seeking among vulnerable groups requires a deep understanding of various interconnected factors this study, factors affecting individuals’ attitudes toward seeking both psychological and psychiatric support were examined. As a result of the study, the research questions were answered. It is related that individuals receive psychological and psychiatric support. It was determined that sociodemographic, individual, and social factors affected both individuals’ attitudes toward seeking psychological support and their attitudes towards seeking psychiatric support.

Promoting mental health literacy, education, training of mental health professionals, and widespread use of mental health services is crucial, especially for at-risk populations. It is suggested to encourage seeking professional psychological or psychiatric support and to facilitate access to these services. It should be ensured that individuals have access to these services in family physicians’ offices, it may even be made obligatory to have a psychologist on staff in institutions above a certain size or based on the characteristics of the institution, and performing routine assessments for the mental health of personnel at certain intervals may be promoted.

Various programs to encourage seeking psychological support should be developed, particularly for men, who are more resistant to seeking psychological support. It is suggested to inform the public through public service ads that state that seeking psychological support is not something to be ashamed of, to provide free psychological support services to individuals who are unemployed for a certain time, to increase the level of education surrounding the issue to raise awareness among society in every respect, and to reduce the use of alcohol and tobacco products by implementing necessary policies.
